# Comparison of electron injection and recombination on TiO_2_ nanoparticles and ZnO nanorods photosensitized by phthalocyanine

**DOI:** 10.1098/rsos.180323

**Published:** 2018-07-11

**Authors:** K. Virkki, E. Tervola, M. Ince, T. Torres, N. V. Tkachenko

**Affiliations:** 1 Laboratory of Chemistry and Bioengineering, Tampere University of Technology, PO Box 541, 33101 Tampere, Finland; 2 Departamento de Química Orgánica, Universidad Autónoma de Madrid, Cantoblanco, 28049 Madrid, Spain; 3 Advanced Technology Research and Application Center, Mersin University, Ciftlikkoy Campus, 33343 Mersin, Turkey; 4 Department of Energy Systems Engineering, Faculty of Tarsus Technology, Mersin University, 33480 Mersin, Turkey; 5 Institute for Advanced Research in Chemical Sciences (IAdChem), Universidad Autónoma de Madrid, 28049 Madrid, Spain; 6 IMDEA Nanociencia, C/Faraday, 9, Cantoblanco, 28049 Madrid, Spain

**Keywords:** TiO_2_ nanoparticles, ZnO nanorods, phthalocyanine, semiconductor–organic interface, photo-induced electron transfer

## Abstract

Titanium dioxide (TiO_2_) and zinc oxide (ZnO) semiconductors have similar band gap positions but TiO_2_ performs better as an anode material in dye-sensitized solar cell applications. We compared two electrodes made of TiO_2_ nanoparticles and ZnO nanorods sensitized by an aggregation-protected phthalocyanine derivative using ultrafast transient absorption spectroscopy. In agreement with previous studies, the primary electron injection is two times faster on TiO_2_, but contrary to the previous results the charge recombination is slower on ZnO. The latter could be due to morphology differences and the ability of the injected electrons to travel much further from the sensitizer cation in ZnO nanorods.

## 1. Introduction

Titanium dioxide (TiO_2_) is the most widely used anode material in dye-sensitized solar cell (DSSC) applications [1], and the highest power conversion efficiencies achieved for DSSCs so far were achieved for cells built on a layer of TiO_2_ nanoparticles [2]. There are a few promising alternative materials under development, probably the most well known being ZnO [3]. ZnO has a band gap position very close to that of TiO_2_, but has higher electron mobility. It is naturally abundant and a few inexpensive methods exist to produce nanoparticles, nanorods and nanowires for DSSC applications. However, the power conversion efficiencies of most ZnO-based DSSCs are lower than those of the best TiO_2_-based DSSCs [4]. However, for TiO_2_ and ZnO cells with identical morphologies, the efficiencies of the two anode materials match each other well, though different factors contribute to their efficiencies [5]. This stimulated our research on the primary electron injection, regeneration, recombination and other reactions at the semiconductor–sensitizing dye interfaces of the two materials [6].

A large number of comparative studies carried out with different sensitizers indicated that electron injection is faster on TiO_2_ than on ZnO [7–11]. It has also been demonstrated that photoinduced electron injection itself is not a simple single-step reaction. At first, an electron–cation complex is formed at the interface which later may yield a ‘free electron’ in the conduction band (CB) of the semiconductor [12,13]. The primary photoinduced electron injection from a sensitizer to an anode can be monitored by ultrafast absorption spectroscopy, which is observation of the sensitizer cation formation in most cases. The separation of the electron–cation complex does not change sample absorption in the visible (Vis)–near-IR (NIR) range, but can be studied by ultrafast terahertz spectroscopy (e.g. [12,14]), and it has been shown that the electron leaves the interface faster in the case of TiO_2_ than in ZnO. A characteristic time scale for this process is around a picosecond for the TiO_2_ interface and up to a few hundreds of picoseconds for ZnO.

It should be noted, however, that for both semiconductor materials (TiO_2_ and ZnO) the electron injection is sufficiently fast for most of the studied sensitizers, and the time constant of the charge separation at the interface alone should not lead to a dramatic difference in the efficiency of photocurrent generation. Another clear difference between TiO_2_ and ZnO materials used as photo-anodes is the crystallinity and electronic band structure. Mostly, anatase TiO_2_ nanoparticles are used to fabricate photo-anodes. ZnO nanoparticles are also commonly used for photo-anodes. Also ZnO nanorods with a distinct wurtzite crystal structure are an attractive alternative material not fully exploited yet [3]. ZnO belongs to the group of direct band gap semiconductors, whereas TiO_2_ has a more complex band structure, which also affects the charge separation at the anode surface [13].

The aim of this study was to compare primary photoinduced reactions on TiO_2_ and ZnO anodes. Standard TiO_2_ anatase nanoparticles were used to prepare TiO_2_ anodes. ZnO nanorods were selected as a model system for the ZnO anodes, as these anodes have well-defined crystallinity (wurtzite) and morphology [15]. Based on our previous experience, a special phthalocyanine derivative was selected as the organic sensitizer [16–18]. This phthalocyanine (Pc) has specifically designed bulky peripheral groups which prevent aggregation and eliminate inter-chromophore interactions. An important advantage of the Pc selected for this study is the strong and sharp absorption features of the ground, excited and cation states, which allow reliable and quantitative characterization based on ultrafast transient absorption measurements. Another important feature is picosecond electron injection, which allows the ‘pure’ excited state to be observed with an instrument limited to 100 fs in time resolution.

## 2. Material and methods

### 2.1. Materials

**Figure 1.**
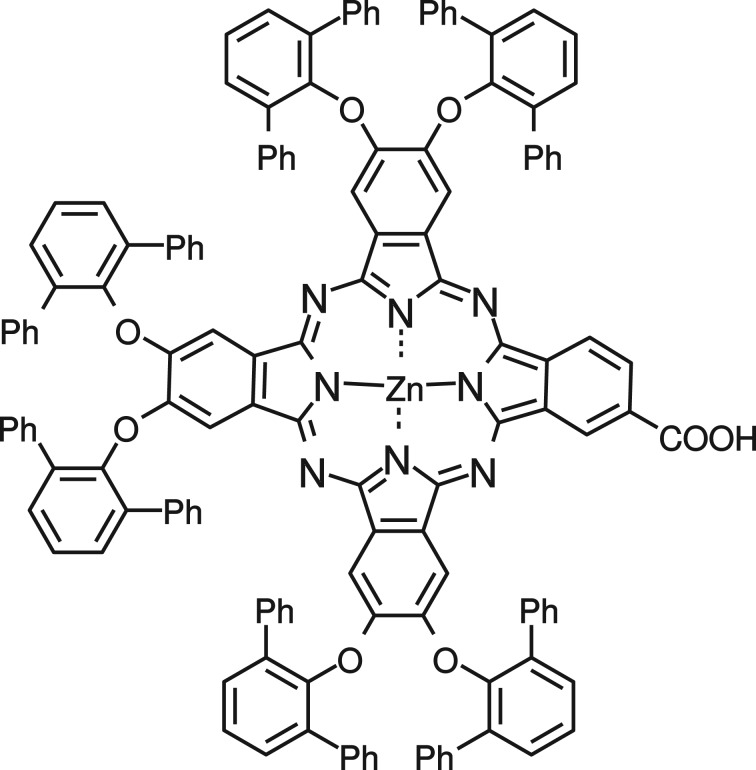
Phthalocyanine (Pc) structure.

The chemical structure of the phthalocyanine (Pc) used is shown in figure 1 and its synthesis is described elsewhere [16]. Ethanol (EtOH) (≥99.5% by mass) was purchased from Altia Plc. Acetonitrile (MeCN), *t*-butanol (*t*-BuOH), ethanolamine, hexamethylene tetramine, 2-methoxyethanol, toluene, zinc acetate dihydrate and zinc nitrate hexahydrate were purchased from Sigma-Aldrich. TiO_2_ nanoparticle paste was purchased from Solaronix (Ti-Nanoxide T/SP) and from Dyesol (18NR-T). Fluorine–tin oxide (FTO)-coated glass substrates (TEC7) were purchased from Sigma-Aldrich and cleaned as described in [18].

### 2.2. Samples

**Figure 2.**
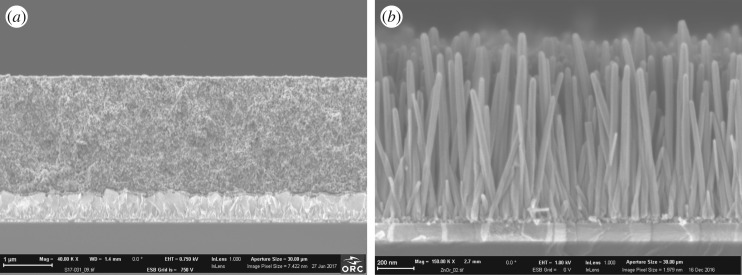
SEM cross-section images of (*a*) a TiO_2_|Pc sample (the lighter-coloured layer in the bottom is FTO with a thin compact layer; the scale bar is 1 μm) and (*b*) a ZnO|Pc sample (the lighter-coloured layer in the bottom is ITO with a thin seed ZnO layer on top; the scale bar is 200 nm).

Previously developed and tested procedures were used to prepare nanostructures [15,19–21]. In brief, TiO_2_ nanoparticle (anatase, average size 20 nm) films were prepared by spin-coating on FTO glasses pre-coated by a thin TiO_2_ compact layer [19,20]. The thickness of the TiO_2_ nanoparticle layer was determined by scanning electron microscope (SEM) (figure 2*a*) and was typically 2–2.3 μm. ZnO nanorod photo-anodes were prepared using a hydro-thermal method on indium–tin oxide (ITO)-coated glasses following the procedure described previously [15,18]. The typical length of the nanorods was 800–1000 nm and diameters were around 50 nm (figure 2*b*). For the studied samples, the specific surface area of the ZnO nanorod samples was estimated to be approximately 10 times smaller than that of the TiO_2_ samples.

Self-assembled monolayers (SAMs) of Pc were deposited by dissolving Pc in a mixture of BuOH : MeCN 1 : 1 (by volume) at a concentration of 0.03–0.1 mM, immersing the substrates into solution for 30 min and washing away the phys-adsorbed excessive Pc with pure solvent, as described elsewhere [18].

### 2.3. Instruments

Absorption spectra of the samples were measured using a Shimadzu UV-3600 UV-VIS-NIR spectrophotometer. The sample morphology was investigated using a field emission SEM (FE-SEM; Carl Zeiss Ultra 55). Ultrafast transient absorption (TA) responses of the samples were measured using a pump–probe system described previously [20,22]. Briefly, samples were excited at 695 nm (pump) by roughly 100 fs pulses at a repetition rate of 1 kHz (Libra F, Coherent Inc., coupled with Topas C, Light Conversion Ltd). White continuum probe pulses were generated by a small fraction of fundamental pulses produced by the generator (Libra F) focused on a sapphire crystal. The time-resolved transient absorption spectra were recorded using an ExciPro TA spectrometer (CDP, Inc.) in two wavelength ranges: 460–770 nm and 850–1050 nm. Home-developed software (decfit.py) was used to process and analyse the pump–probe measurements. The program carries out group velocity dispersion compensation, convolution with an instrument response function and a global data fit to a sum of exponential, stretched exponential and distributed decay functions [23].

## 3. Results

### 3.1. Samples

**Figure 3.**
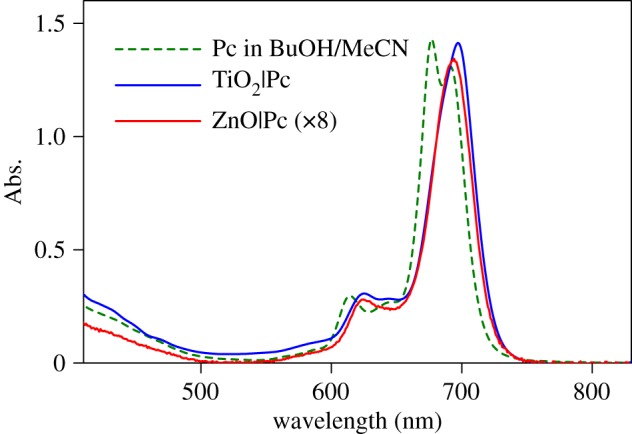
Absorption spectra of TiO_2_|Pc and ZnO|Pc samples after subtracting substrate spectra, and Pc in mixture of BuOH : MeCN. The spectrum of ZnO|Pc sample was multiplied by eight to match the scale.

Absorption spectra of the Pc-sensitized samples are presented in figure 3 together with the solution spectrum. The absorption spectra of the substrates, TiO_2_ nanoparticle film and ZnO nanorod array were subtracted; therefore, the spectra shown represent absorption of Pc SAMs. Owing to the thickness and morphology differences, the TiO_2_ samples had an approximately 10 times higher surface specific area than the ZnO samples [15]. Therefore, to compare Pc spectra in TiO_2_ and ZnO, the spectrum of the ZnO|Pc sample was multiplied by 8. The resulting absorption spectra of the Pc SAMs on both ZnO and TiO_2_ are reasonably close to each other in shape, and show no evidence of Pc aggregation. One may notice a red shift (by roughly 8 nm) and some differences in the shapes of the spectra in the Q-band area (650–710 nm region) of the SAMs compared with the solution. However, the band width is the same 37 nm (full width half-maximum) in both cases, which indicates no aggregation effect on the spectra of the SAMs.

### 3.2. Transient absorption spectroscopy

**Figure 4.**
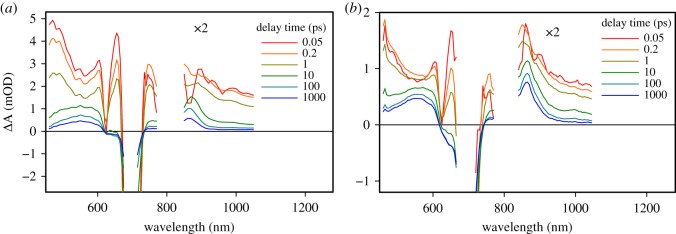
Time-resolved transient absorption spectra of (*a*) TiO_2_|Pc and (*b*) ZnO|Pc samples at a few selected delay times. The data were corrected for the group velocity dispersion.

The photoinduced reactions in the samples were studied by measuring the transient absorption responses (pump–probe method) in two wavelength ranges, 460–770 nm and 850–1050 nm, with excitation at 695 nm. The time-resolved differential absorption spectra of TiO_2_|Pc and ZnO|Pc samples at selected delay times are presented in figure 4. At a qualitative level, the spectra at a short delay time, e.g. at 0.2 ps, can be attributed to the singlet excited state of Pc formed immediately after the excitation, and at a longer delay time, e.g. at 1 ns, they can be attributed to the Pc cation [24]. The responses of two samples in figure 4 have much in common in terms of spectral shapes, though lifetimes and relationships between different spectral features are clearly different.

**Figure 5.**
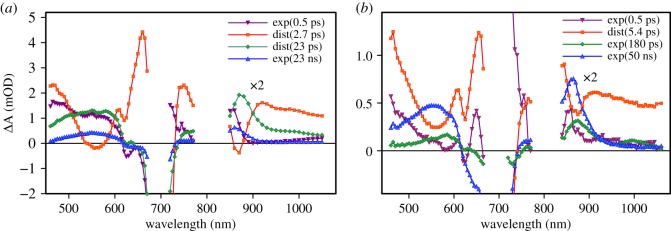
Transient absorption component spectra of Pc on (*a*) TiO_2_ nanoparticles and (*b*) ZnO nanorods. The fit model consisted of the sum of the exponential and distributed decays denoted as exp(…) and dist(…), respectively, with characteristic time constants indicated in brackets. The NIR part of the spectra (*λ* > 800 nm) is magnified two times.

The transient absorption response of the ZnO|Pc sample can be fitted reasonably well by a sum of three exponential and one distributed decay functions as discussed in [18]. The resulting decay-associated component spectra are presented in figure 5*b*. These are spectra of the transient absorption responses associated with corresponding fit functions, exponential and distributed decays denoted as ‘exp(…)’ and ‘dist(…)’, respectively. To obtain an equally good fit of the TiO_2_|Pc data, a combination of two exponents and two distributed decays had to be used (figure 5*a*). The spectral shapes are highly similar for the two sets of the component spectra, even though the relative intensities and corresponding time constants are different.

**Figure 6.**
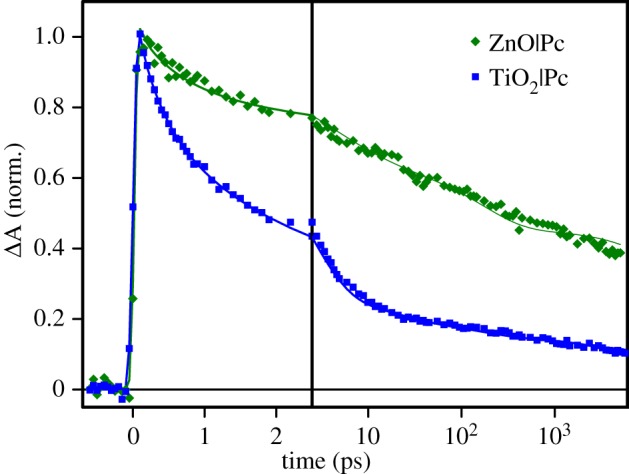
Transient absorption decays at 865 nm of Pc on TiO_2_ and ZnO. The symbols are the measured data points and the solid lines are the fits.

The wavelength of most different decay profiles of the transient absorptions of TiO_2_ and ZnO samples is 865 nm. The normalized decays at 865 nm are shown in figure 6. This figure illustrates also the fit quality (solid lines): there were no regular deviations of the fits from the data through the whole spectrum range and typical sigma-values were 0.01–0.02 mOD.

## 4. Discussion

### 4.1. Long-lived charge separation

In both cases, the longest-lived components (figure 5) have decay times much longer than the maximum delay time available to the instrument (6 ns); therefore, the calculated decay time values are not accurate. However, the spectra of the components are very much alike and show a relatively narrow absorption band at 865 nm and a broad absorption band around 560 nm in addition to strong bleaching of the Q-band around 700 nm. These features are typical for the Pc cation, Pc^+^ [18,24,25]. The relative intensities of the long-lived components are rather different in the two samples, being much stronger in the ZnO|Pc sample.

### 4.2. Electron injection

Another component with clear and distinct features is the distributed decay component with an average time constant of 2.7 ± 0.2 ps in the case of the TiO_2_|Pc sample and 5.4 ± 0.7 ps for the ZnO|Pc sample. It has a sharp band at 650 nm, a relatively sharp negative peak at 870 nm and a broad negative peak around 560 nm. These features are consistent with transition from the singlet excited state to the Pc cation, which is caused by electron injection to TiO_2_,

4.1 $${\text{TiO}}_{2}|{\text{Pc}}^{*}\overset{\tau_{\mathrm{inj}}}{\to }{\text{TiO}}_{2}^{-}|{\text{Pc}}^{+}.$$

The distributed decay model operates with three parameters [23]: the average (or non-disturbed) time constant (*τ*_inj_) indicated for the corresponding decay components in figure 5, the width of the free and/or reorganization energy distribution (△E), and the sensitivity factor (*a*). The time constant is approximately two times larger for the ZnO|Pc sample, but the other two parameters are nearly identical for the two samples, △E=0.022±0.004 eV and *a* = 1.0 ± 0.2 for TiO_2_|Pc and △E=0.028±0.007 eV and *a* = 1.2 ± 0.3 for ZnO|Pc. This means that the electron injection is two times faster in the TiO_2_|Pc sample, but otherwise the mechanism of electron transfer is the same.

There is a fast 0.5 ps component in the response of both samples. It has an effect of a minor absorption decay in the visible part of the spectrum and some reshaping around 850 nm, but it does not lead to the formation of an intermediate state with new distinct spectral properties (figure 4). This component is tentatively attributed to the thermal relaxation of the singlet excited state and will not be discussed further.

### 4.3. Fast charge recombination at the interface

The last component to discuss is that of the 180 ps time constant in the case of the ZnO|Pc sample and the 23 ps average time constant (distributed decay component) for the TiO_2_|Pc sample. The spectral shapes of this component are rather close for the two samples but the relative intensities are different. It is a minor component for the ZnO|Pc sample and one of the strongest for the TiO_2_|Pc sample. Furthermore, the spectra of these components resemble those of the longest-lived component, with differences mainly seen in the shape of the broad band in the 500–600 nm range and an absorption shoulder (TiO_2_|Pc) or a minor band (ZnO|Pc) in the 950–1050 nm range. The latter can be associated with a Pc anion, which has two characteristic bands: one is close to 1000 nm in the NIR and the other is at 590 nm in the visible part of the spectrum, as was observed for ZnO|Pc covered with a hole-transporting material (electron donor) [18]. The appearance of a Pc anion could be explained by the partial aggregation of the Pc molecules and an intra-aggregate charge separation in the process ${\text{Pc}}^{*}-\text{Pc}\to {\text{Pc}}^{+}-{\text{Pc}}^{-}$. This could be a reasonable explanation in the case of the ZnO|Pc sample, though for the ZnO|Pc sample it is a minor process in any case. However, in the case of the TiO_2_|Pc sample, there is no band at 1000 nm and the component shape in the 500–620 nm range does not match that of the Pc anion, having a broad band in the 500–600 nm range rather than a relatively narrow anion band at 590 nm. It is more reasonable to attribute this component to a Pc cation, which leaves a question on the origin of two Pc cations with drastically different lifetimes, 23 ps and 23 ns (TiO_2_|Pc) and minor differences in the spectral shapes.

A typical interpretation of a very diverse decay time of one and the same state is inhomogeneity of the Pc arrangement on the TiO_2_ surface. However, this can also be ruled out for two reasons. Firstly, the bulky peripheral groups and rigid core of Pc makes a diverse conformation distribution unlikely. Secondly, the same diversity could be expected for both semiconductor substrates, but in the case of ZnO the fast charge recombination (180 ps) is a minor process, whereas in the case of TiO_2_ it is almost the dominating process.

### 4.4. Interfacial charge transfer complex

There are reports on a two-step charge separation at the semiconductor–organic interface [9,11,12,14]. In the first step, a coupled electron–hole pair is formed with the electron in the CB but electrostatically bound to the sensitizer cation, Pc^+^. This state is also termed the interfacial charge-transfer complex (ICTC) [13]. It may recombine or the electron may leave the interface and become a ‘free’ carrier in the CB. In the spectral range studied, the measured transient absorption response is dominated by the response of the Pc sensitizer, a cation in both the coupled and non-coupled Pc^+^ states. Therefore, electron migration from the close proximity of the Pc^+^ to the bulk of the semiconductor is expected to have only a minor effect on the transient absorption spectrum shape, which is consistent with the minor spectral differences of the two long-lived components in figure 5.

Taking this two-step charge transfer as a working hypothesis, one could conclude that there is a relatively large probability of charge recombination of the ICTC in the case of TiO_2_|Pc, as the molar absorption coefficients of the ‘free’ cation Pc^+^ and cation in the ICTC must be almost the same. The gradual decay of the signal at 865 nm within a few tens of picoseconds (figure 6) points to the complex recombination but not to the electron shift to the bulk of TiO_2_. However, this conclusion contradicts previous studies. First of all, terahertz spectroscopy of similar TiO_2_ samples suggests that the ICTC dissociates by yielding free electrons in the CB within 1 ps [12,14], much shorter than the 23 ps time constant discussed here. On the contrary, for ZnO samples, this dissociation time is extended to 100 ps and agrees reasonably well with the 180 ps reported here. Secondly, Chegui and co-workers [13] have shown that the fates of the injected electron are very different in TiO_2_ and ZnO due to the different electronic band structures, a direct band gap in ZnO and an indirect one in TiO_2_, and ICTC is formed at the ZnO|Pc interface but not at the TiO_2_|Pc interface. Therefore, our results are consistent with an intermediate ICTC complex in the ZnO|Pc sample only.

The 23 ps time constant corresponds to an electron diffusion length of a few tens of nanometres in bulk TiO_2_, roughly the size of a nanoparticle. This leads us to the assumption that the fast charge recombination is associated with the probability of the electron remaining in the same nanoparticle into which it was injected. The free electron and Pc^+^ cannot recombine as soon as the electron jumps to another nanoparticle, and 23 ps is an average time for the free electron to move from one TiO_2_ nanoparticle to another. The characteristic size, the length, of ZnO nanorods is much larger and the carrier mobility is much higher in ZnO, therefore the same fast recombination mechanism in not efficient in ZnO| samples since free electrons move quickly away from the recombination centre, Pc^+^. This assumption has no direct proof, however.

### 4.5. Dynamics of the Pc cation on TiO_2_ and ZnO surfaces

The band at 865 nm is a characteristic feature of the Pc cation. Unfortunately, the singlet excited state of Pc also has relatively high absorption at this wavelength, which is the reason why the electron injection (reaction (4.1)) does not lead to an absorption rise at this wavelength in the first few picoseconds of electron injection and cation formation. Nevertheless, at relatively long delay times when the singlet state has already relaxed, e.g. >10 ps, the signal at 865 nm can be used as an indicator of the [Pc^+^] population. Comparison of the transient absorption decays at this wavelength is shown in figure 6. Obviously, the charge recombination at the TiO_2_|Pc interface is faster than that at the ZnO|Pc interface, and the most drastic drop of the signal at 865 nm (TiO_2_|Pc sample) takes place within the first 10 ps after the excitation.

A very simplified reaction scheme for both samples is

4.2 $$\begin{array}{llcll} \text{Pc*} & \overset{\tau_{\mathrm{inj}}}{\to } & e^{-}|{\text{Pc}}^{+} & \overset{\;\tau_{l}\;}{\to } & {\text{Pc}}^{+}\\ & & \;\;\;\;\;\;\;↓\tau_{\mathrm{int}} & & ↓\tau_{\mathrm{cr}}\\ & & \;\;\;\;\;\;\;\text{Pc} & & \text{Pc} \end{array}$$

where *e*^−^|Pc^+^ is the ICTC or any other fast disappearing charge separated state, *τ*_int_ is the time constant of this state charge recombination, *τ*_l_ is the time constant to form a ‘long-lived’ charge transfer state, and *τ*_cr_ is the final recombination time constant of Pc^+^. Experimentally observable time constants are *τ*_int_, *τ*_cr_ and *τ*_s_ = (*τ*^−1^_int_ + *τ*^−1^_l_)^−1^, which are the time constants of the picosecond, 20–200 ps, and nanosecond components in figure 5.

To model the process dynamics, the fit results at 865 nm will be used. Then, we will assume that:

(i) the picosecond component is solely due to the relaxation of the singlet excited state, Pc*; thus the average time constants for the singlet state relaxation are *τ*_inj_ = 2.4 ± 0.2 ps and 5.4 ± 0.7 ps for the TiO_2_|Pc and ZnO|Pc samples, respectively;
(ii) only the excited state, Pc*, and cation, Pc^+^, contribute to the transient absorption at this wavelength, and absorption of two cations in scheme (4.2) is the same;
(iii) immediately after excitation only Pc* is formed, which means that the time resolution of the instrument is sufficient to resolve the cation formation and it has zero population at zero time.

Within this model, the population decay of the excited state is

**Figure 7.**
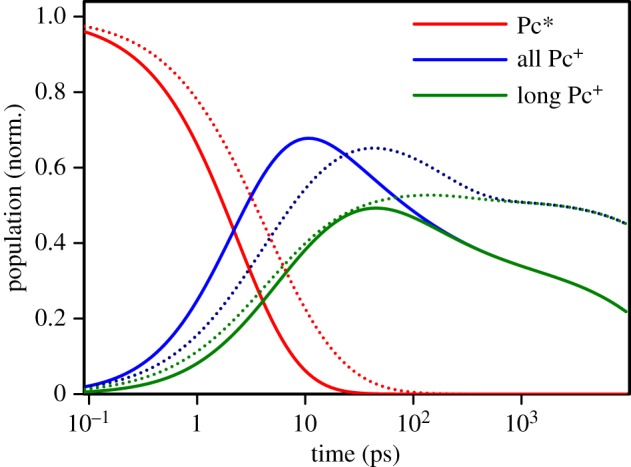
Modelled dynamics of the singlet excited state, Pc* and cation, Pc^+^, populations in the TiO_2_|Pc (solid lines) and ZnO|Pc (dotted lines) samples. The population of Pc cations was calculated for all Pc^+^ and for long-lived Pc^+^, see text for the details.

4.3 $$[{\text{Pc}}^{*}](t)=\text{dist}(t,\tau_{\mathrm{inj}},△E,a),$$

where the function dist() is computed as presented in [23], and it depends on the ‘non-disturbed’ or average time constant, *τ*_inj_, the energy distribution width, △E, and the sensitivity factor, *a*. The population decay of the singlet excited state is shown in figure 7 by the red lines.

The time profile of the total cation population (‘all Pc^+^’ in figure 7, blue lines) can be computed as the difference between the normalized decays at 865 nm ($△A_{\lambda =865\;\text{nm}}(t)$) and [Pc*](*t*), though this does not give an ‘absolute’ value of the Pc^+^ population since the ratio of molar absorbances of Pc^+^ and Pc* is not known. In other words, the sum ‘Pc*’ and ‘all Pc^+^’ in figure 7 is the normalized measured decay at 865 nm, or the fits shown in figure 6.

Next, the proportion in the branching reactions of the *e*^−^|Pc^+^ state can be evaluated from the relative intensities of the decay components with the time constants 23 ps and 23 ns (in the case of the TiO_2_|Pc sample), which gives

4.4 $$\tau_{\mathrm{int}}=\tau_{c}\frac{a_{\mathrm{int}}}{a_{l}+a_{\mathrm{int}}}$$

and

4.5 $$\tau_{l}=\tau_{c}\frac{a_{l}}{a_{l}+a_{\mathrm{int}}},$$

where *τ*_*c*_ is the measured time constant of the middle decay component (23 ps for TiO_2_). The green lines in figure 7 show cation populations minus the population of the *e*^−^|Pc^+^ states; figure 7 also presents the dynamics of the ‘long-lived’ cations.

The most reliable part here is the separation of the singlet, Pc*, and cation, Pc^+^, as it is based on sharp spectral differences between the two states. Division on the ‘short’- and ‘long’-lived Pc cations (states *e*^−^|Pc^+^ and Pc^+^ in scheme (4.2)) is rather phenomenological, but it reflects the experimental observation that the cation Pc^+^ decay has fast, 23 ps, and slow, 23 ns, components with approximately two-thirds accumulated in the fast decay in the case of the TiO_2_|Pc sample. This is not the case for the ZnO|Pc sample, for which the fast decay is the minor component. This model suggests that the relative population of Pc^+^ is higher in the ZnO|Pc sample at delays longer than 20 ps. Although it has to be noted that in complete solar cells the photon-to-current conversion efficiency depends also on how fast the holes from the sensitizer are transferred to the electrolyte in liquid cells or hole-transporting material in solid-state cells. If this process is sufficiently fast, taking not more than a few tens of picoseconds, both systems, TiO_2_|Pc and ZnO|Pc, are expected to be equally efficient. This conclusion is in line with the comparative study of Chandiran *et al.* [5].

## 5. Conclusion

Figure 7 summarizes the discussion of the differences between the TiO_2_ nanoparticles (anatase) and ZnO nanorods (wurtzite) as the electron-collecting materials photosensitized by a Pc. The experimental results suggest that the primary electron injection is faster in the TiO_2_ sample, in agreement with numerous previous observations. But the charge recombination is also faster in TiO_2_. Furthermore, a relatively large number of the injected electrons recombine with the Pc cation on the semiconductor surface within a few tens of picoseconds in the case of TiO_2_, but not in ZnO. Overall, the yield of Pc cations and thus the yield of the electrons injected into the anode is higher in ZnO at delays longer than 20 ps with the difference increasing with the delay time. This advantage of ZnO nanorods may be due to two factors: higher electron mobility in ZnO than in TiO_2_, and a longer travelling distance in ZnO nanocrystals and nanorods, which helps to separate electrons and surface cations, Pc^+^, more efficiently in the case of ZnO.

## Data accessibility

This article has no additional data.
